# Treatment of Whole-Plant Corn Silage With Lactic Acid Bacteria and Organic Acid Enhances Quality by Elevating Acid Content, Reducing pH, and Inhibiting Undesirable Microorganisms

**DOI:** 10.3389/fmicb.2020.593088

**Published:** 2020-12-04

**Authors:** Fu-gui Jiang, Hai-jian Cheng, Dong Liu, Chen Wei, Wen-juan An, Ya-fang Wang, Hai-tao Sun, En-liang Song

**Affiliations:** ^1^Institute of Animal Science and Veterinary Medicine, Shandong Academy of Agricultural Sciences, Jinan, China; ^2^Shandong Key Lab of Animal Disease Control and Breeding, Jinan, China; ^3^Shandong Provincial General Station of Animal Husbandry, Jinan, China; ^4^College of Life Sciences, Shandong Normal University, Jinan, China

**Keywords:** corn, microbial community, silage, lactic acid bacteria, organic acid

## Abstract

We investigated the variation in microbial community and fermentation characteristics of whole-plant corn silage after treatment with lactic acid bacteria (LAB) and organic acids. The fresh corn forages were treated with a combination of *L. acidophilus* and *L. plantarum* (10^6^ CFU/g fresh material) or a 7:1:2 ratio of formic acid, acetic acid, and propionic acid (6 mL/g fresh material) followed by 45 or 90 days of ensiling. Silages treated with LAB showed increased lactic acid content and decreased pH after 45 days. Although treatment with LAB or organic acids decreased the common and unique operational taxonomic units, indicating a reduction in microbial diversity, the relative abundance of *Lactobacillus* was elevated after 45 and 90 days compared with control, which was more distinct in the organic acid groups. Moreover, we found higher levels of acetic acid and increased abundance of *Acetobacter* in silages treated with organic acids whereas undesirable microorganisms such as *Klebsiella*, *Paenibacillus*, and *Enterobacter* were reduced. In summary, the quality of corn silages was improved by LAB or organic acid treatment in which LAB more effectively enhanced lactic acid content and reduced pH while organic acid inhibited the growth of undesirable microorganisms.

## Introduction

Corn silage is a highly nutritious forage for livestock and has a higher yield and energy density as well as lower cost compared with other forage sources (Armstrong et al., 2008). The fermentation process of corn silage is dominated by many kinds of microbes and aims to not only preserve the corn but also to achieve a high-quality silage and improve animal performance (Muck et al., 2018). The fermentation mechanism is a complex and dynamic process that affected by many factors such as anaerobic conditions in the silo, levels of water-soluble carbohydrates (WSC), abundance of epiphytic bacteria, amount of dry matter, and buffering capacity of the materials (Weinberg and Muck, 1996). Consequently, silage quality needs to be effectively maintained by using additives. Currently, a wide variety of silage additives have been developed to improve fermentation including fermentation stimulants and inhibitors, chemicals, and enzymes (McDonald et al., 1991).

Lactic acid bacteria (LAB) are commonly used as stimulants to improve silage fermentation (Muck et al., 2018). However, past studies have reported that an effectiveness of LAB was influenced by several factors including forage type, LAB species and quantity, and other ensilage management practices (Weinberg and Muck, 1996; Blajman et al., 2018). A meta-analysis of 130 articles revealed that LAB inoculants improved the fermentation of grass and legume silages, but did not affect the fermentation of corn, sorghum, and sugar cane silages (Oliveira et al., 2017). Interestingly, inoculated silage with LAB enhanced milk production based on a meta-analysis of 31 lactating dairy cow studies and the increased effects were not affected by forage type and LAB species (Oliveira et al., 2017; Muck et al., 2018). The insignificant effect on corn silage fermentation may be caused by the presence of higher levels of WSC and epiphytic bacteria as well as its low buffering capacities (Weinberg and Muck, 1996). In addition, the improved animal performance by LAB may be attributed to the inhibition of detrimental microbes and interactions with rumen microbes (Weinberg and Ashbell, 2003; Ellis et al., 2016). Thus, profiling of the silage microbial community may improve our understanding of silage formation and preservation.

Organic acids such as formic acid, acetic acid or propionic acid have been applied as inhibitors to improve silage fermentation. Formic acid reduces pH by direct acidification and inhibits undesired spoilage bacteria such as enterobacteria and aerobes, thus providing optimal conditions for rapid LAB growth and domination in the silage (Kung et al., 2003). However, formic acid was less effective for the inhibition of yeasts and molds (Henderson et al., 1972) and even improved the growth of some undesirable organisms (McDonald et al., 1991). In contrast, acetic and propionic acid direct inhibit both yeasts and molds and improve the aerobic stability of silages (Kung et al., 2003). A mixture of these organic acids has been used to achieve the combined benefits for silage production. However, previous studies mostly investigated the effects of organic acids on fermentation quality and aerobic stability (Adesogan and Salawu, 2004; Saarisalo et al., 2006; Zhang et al., 2019) and few studies have been conducted regarding changes in microbial communities.

In the past, molecular techniques such as denaturing gradient gel electrophoresis (DGGE), real-time polymerase chain reaction (RT-PCR), and ribosomal intergenic spacer analysis (RISA) have been used to assess the microbial community in silages (Stevenson et al., 2006; Brusetti et al., 2011; Li and Nishino, 2011). However, these approaches offer limited insight regarding the overall properties of these communities. More recently, next-generation high-throughput sequencing (NGS) has been used to investigate microbial communities in *Miscanthus floridulus* (Li et al., 2015), soybean (Ni et al., 2017), corn stalk (Zhang et al., 2018), and corn silage (Keshri et al., 2018). Although previous studies have evaluated the effects of chemical additive on silage microbiome (Ren et al., 2018; Zhang et al., 2020), the microbial community in corn silage treated with mixed organic acids has not yet been investigated. Therefore, this study aimed to compare the impact of mixed organic acids with LAB supplementation on microbial communities in corn silage using NGS.

## Materials and Methods

### Corn Harvest and Silage Treatment

A corn hybrid (Denghai 605, Shandong Denghai Seeds, China) was planted on 12 July 2018 at the testing site of Shandong Academy of Agricultural Sciences (117°58′E, 37°63′N). The crop was harvested on 16 September 2018 and cut into 20-mm long segments using a chopper (FS-690, Zili, China). The dry matter (DM) of the corn material before ensiling was 32.74%. The chemical and microbial composition of corn material before ensiling was listed in Table 1. The chopped corn was mixed and divided into three treatment groups: untreated (CON), LAB treatment (LAB), and organic acid treatment (A). The LAB additive was a commercial product consisting of a combination of *L. acidophilus* and *L. plantarum* (Taiwan Yaxin Biotechnology, China), which was inoculated at a concentration of 10^6^ CFU/g of fresh material. The mixed organic acid additive was composed of formic acid (10010118, Sinopharm Chemical Reagent, China), acetic acid (10000218, Sinopharm Chemical Reagent, China), and propionic acid (79-09-4, Damao Chemical Reagent, China) at a ratio of 7:1:2 (v/v) and was applied at a concentration of 6 mL/kg of fresh material. The LAB and mixed organic acids additive were mixed with 6 mL of sterile distilled water and sprayed onto the 1,000 g chopped corn material (only 6 mL of sterile distilled water for CON). The mixed material (1,000 g) was packed into a plastic bag (20 × 30 cm; Deli Group, China) and the air was eliminated by a vacuum sealer (Deli 14886, Deli Group, China). Four replicates for each treatment were made and ensiled for 45 and 90 days, respectively. Thus, a total of 24 silage samples (4 replicates × 3 treatments × 2 ensiling days) were made and then stored in laboratory at room temperature (21–25°C).

**TABLE 1:** Chemical and microbial composition of fresh corn.

Items	Whole plant corn silage
DM (% FM)	32.74 ± 0.44
CP (% DM)	8.28 ± 0.18
NDF (% DM)	39.86 ± 0.35
ADF (% DM)	21.18 ± 1.31
ADL (% DM)	2.78 ± 0.37
Ash (% DM)	4.71 ± 0.10
EE (% DM)	3.08 ± 0.53
WSC (% DM)	10.61 ± 1.23
LAB (log cfu/g FM)	5.47 ± 0.42
Yeast (log cfu/g FM)	6.73 ± 0.73

**DM, dry matter; CP, crude protein; NDF, neutral detergent fiber; ADF, acid detergent fiber; ADL, acid detergent lignin; EE, ether extract; WSC, water soluble carbohydrate; LAB, Lactic acid bacteria; FM, fresh material.**

### Analysis of Chemical Composition, Microbial Composition, and Fermentation Parameters

To determine the chemical composition, samples were incubated at 65°C for 48 h and ground through a 1-mm screen. The analytical dry matter (DM) was analyzed by drying at 105°C for 3 h. The crude protein (CP), ash, ether extract (EE), and acid detergent lignin (ADL) were measured according to the standard procedures of the Association of Official Analytical Chemists (AOAC, 1990). The content of neutral detergent fiber (NDF) and acid detergent fiber (ADF) was detected as described by Van Soest et al. (1991). The water-soluble carbohydrate (WSC) content was determined by colorimetry as described previously (Murphy, 1958). Dry matter recovery was calculated based on the starting and final weights of the fresh and ensiled materials.

For microbial analysis, 20 g of fresh corn material was homogenized in 180 mL sterile water and 10× serially diluted. LAB abundance was determined using the plate count method on de Man, Rogosa, and Sharpe agar (CM361, Oxoid, United States). The plates were anaerobically incubated at 30°C for 48 h using AnaeroPack-Anaero (Mitsubishi Gas Chemical, Japan). Yeast count was quantified on potato dextrose agar (HB0233-12, Qingdao Hope Bio-Technology, China) following a 48 h incubation at 30°C. Colony counts are presented as the number of viable microorganisms in CFU/gram fresh material.

For determination of pH, organic acid and ammoniacal nitrogen (NH_3_–N), 20 g of corn silages was homogenized in 180 mL sterile water followed by filtering with 0.22 μm membrane filters. Then, 100 mL was used to determine pH using a HI991000 pH meter (Hanna Instruments, United States). The organic acid content was measured by high-performance liquid chromatography using an 8.0 mm × 30 cm Shodex RSpak KC-811S-DVB gel C column (Shimadzu, Japan) at 50°C with a mobile phase of 3 mmol/L HClO4, a flow rate of 1.0 mL/min, an injection volume of 5 ml, and an SPD-M10AVP detector (210 nm). Phenol-hypochlorite assay was used to measure the concentration of NH_3_–N as described by Weatherburn (1967).

### Microbiome Analysis

#### DNA Extraction and PCR Amplification

Approximately 50 g of each treatment sample was collected and immediately frozen with liquid nitrogen for bacterial DNA extraction using a DP302-02 DNA extraction kit (Tiangen, China). The concentration and purity of the extracted DNA were determined using a NanoDrop 2000 UV-vis spectrophotometer (Thermo Fisher Scientific, United States). The quality of the extracted DNA was determined by 1% agarose gel electrophoresis (AGE).

The PCR amplification of the V3-V4 16S rRNA region was carried out with 338F (5′-ACTCCTACGGGAGGCAGCAG-3′) and 806R (5′-GGACTACHVGGGTWTCTAAT-3′) primers. The PCR products were isolated by 2% AGE and further purified by DNA Gel Extraction Kit (Axygen, United States) and quantified by QuantiFluor^TM^-ST (Promega, United States).

#### Sequencing and Analysis

Equal amounts of purified PCR products were pooled and paired-end sequenced (2 × 300 bp) on an Illumina MiSeq platform (Illumina, United States). The paired-end reads were quality-filtered by Trimmomatic software as described previously (Bolger et al., 2014) to eliminate the ambiguous bases and poor-quality sequences with an average quality score of <20. After the pre-processing, paired-end reads were merged using FLASH V1.2.11 (Reyon et al., 2012) with overlap sequences in the range of 10 – 200 bp and a maximal mismatch of <20%. Sequences were further denoised by QIIME V1.8.0 (Caporaso et al., 2010) to remove ambiguous, homologous, or <200 bp sequences and retain the reads with 75% of the bases above Q20. Furthermore, chimeric sequences were also detected and removed. UPARSE V7.0.1090 was applied for operational taxonomic unit (OTU) clustering based on a 97% similarity threshold (Edgar, 2013). To analyze the taxonomy, the Silva (SSU132) 16S rRNA database was used for alignment of the 16S rRNA sequences based on the RDP classifier algorithm V2.2 (Wang et al., 2007) with a 70% confidence threshold. Alpha diversity indices (Ace, Shannon, Chao1, Simpson, and Good’s coverage) were conducted by MOTHUR V1.30.1. Principal coordinates analysis (PCoA) and analysis of similarities (ANOSIM) were conducted by QIIME V1.7.0.

#### Statistical Analysis

Data on the chemical composition and fermentation characteristics were analyzed by the mixed model procedure (PROC MIXED) in SAS V9.1. All data are presented as least-squares mean. The differences among the least-squares means were compared using PDIFF with Tukey’s adjustment. The effects of the factors were considered significant at *P* ≤ 0.05 unless otherwise noted and trends were recognized at 0.05 < *P* ≤ 0.10.

## Results

### Chemical and Microbial Composition of Fresh Corn

After processing the fresh material, the DM was 32.74% of the original weight (Table 1). The CP, NDF, ADF, EE, and WSC were 8.28, 39.86, 21.18, 3.08, and 10.61%, respectively. Moreover, the LAB and yeast counts were 5.47 and 6.73 log CFU/gram fresh material, respectively.

### Chemical Composition of Corn Silage

LAB treatment showed comparable DM content and recovery with controls, although that of organic acid treatment was significantly lower (*P* < 0.05, Table 2). The NDF, ADF, and WSC levels between LAB and organic acid-treated corn silage were similar and significantly reduced compared with control (P < 0.05). The CP, EE, ADL, and ash levels were not affected by the treatments. The ensiling duration did not affect the chemical composition of the silages.

**TABLE 2:** Chemical composition of corn silage treated with LAB and organic acids after 45 and 90 days of ensiling.

Items	45 days			90 days				*P*		
				
	CON	LAB	A	CON	LAB	A	SEM	T	D	T × D
DM (% FM)	30.80*^ab^*	31.29*^ab^*	30.76*^ab^*	30.80*^ab^*	31.52^*a*^	30.58^*b*^	0.120	0.029	0.936	0.745
DM recovery (%)	92.18*^ab^*	93.66^*a*^	92.07*^ab^*	92.18*^ab^*	94.35^*a*^	91.54^*b*^	0.356	0.011	0.926	0.676
CP (% DM)	8.16	8.37	8.43	8.36	8.22	8.52	0.060	0.351	0.694	0.510
NDF (% DM)	35.14*^ab^*	34.48^*b*^	34.24^*b*^	36.96^*a*^	34.86^*b*^	34.49^*b*^	0.305	0.046	0.149	0.434
ADF (% DM)	20.67^*a*^	19.75*^ab^*	19.29^*b*^	20.53*^ab^*	19.63*^ab^*	19.67*^ab^*	0.182	0.033	0.894	0.780
ADL (% DM)	1.66	1.53	1.52	1.72	1.60	1.67	0.057	0.679	0.460	0.951
Ash (% DM)	4.40	4.91	4.86	5.01	4.75	4.95	0.078	0.568	0.254	0.127
EE (% DM)	4.00	4.11	4.19	4.29	4.14	4.10	0.056	0.987	0.556	0.435
WSC (% DM)	5.37^*a*^	4.09^*b*^	4.25^*b*^	5.70^*a*^	3.96^*b*^	4.57^*b*^	0.159	<0.001	0.376	0.534

*Values in the same row with different superscript letters differ at P < 0.05.*

*CON, corn silage treated without inoculant; LAB, corn silage treated with lactic acid bacteria inoculant; A, corn silage treated with organic acids. T, treatments effect; D, ensiling days effect; T × D, interaction effect between treatments and ensiling days.*

*DM, dry matter; CP, crude protein; NDF, neutral detergent fiber; ADF, acid detergent fiber; ADL, acid detergent lignin; EE, ether extract; WSC, water soluble carbohydrate.*

### Fermentation Characteristics of Corn Silage

The pH of silages decreased with prolonged ensilage with simultaneous increases in acetic acid concentration (*P* < 0.05, Table 3). Interestingly, this increase in acetic acid concentration after prolonged ensilage was only observed in the organic acid groups and not in the control and LAB-treated corn silage. The acetic and propionic acid concentrations were similar between control and LAB groups and were lower than in the organic acid groups (*P* < 0.05). Lactic acid content in the organic acid-treated corn silage was higher compared with control groups (*P* < 0.05; 57.14 vs. 49.59%) but lower than LAB-treated corn silage (*P* < 0.05; 57.14 vs. 62.58%).

**TABLE 3:** Fermentation characteristics of corn silage treated with LAB and organic acids after 45 and 90 days of ensiling.

Items	45 days			90 days				*P*		
				
	CON	LAB	A	CON	LAB	A	SEM	T	D	T*D
pH	3.51^*a*^	3.47^*b*^	3.48*^ab^*	3.43^*c*^	3.46*^bc^*	3.43^*c*^	0.01	0.513	<0.001	0.012
Lactic acid (g/kg DM)	44.44^*b*^	66.63^*a*^	58.47^*a*^	54.74*^ab^*	58.53^*a*^	55.81*^ab^*	1.92	0.009	0.960	0.063
Acetic acid (g/kg DM)	11.49^*c*^	11.94*^bc^*	18.37^*b*^	12.37*^bc^*	14.12*^bc^*	29.40^*a*^	1.50	<0.001	0.010	0.042
Propionic acid (g/kg DM)	0.07^*b*^	0.10^*b*^	2.62^*a*^	0.08^*b*^	0.05^*b*^	2.74^*a*^	0.26	<0.001	0.720	0.590
Butyric acid (g/kg DM)	0.01	0.02	0.02	0.02	0.03	0.01	<0.01	0.643	0.870	0.599
NH_3_-N (g/kg DM)	3.00	2.70	2.84	2.68	2.43	2.64	0.09	0.507	0.180	0.970

*Values in the same row with different superscript letters differ at P < 0.05.*

*CON, corn silage treated without inoculant; LAB, corn silage treated with lactic acid bacteria inoculant; A, corn silage treated with organic acids. T, treatments effect; D, ensiling days effect; T × D, interaction effect between treatments and ensiling days.*

### Microbial Community in Corn Silage

**FIGURE 1:**
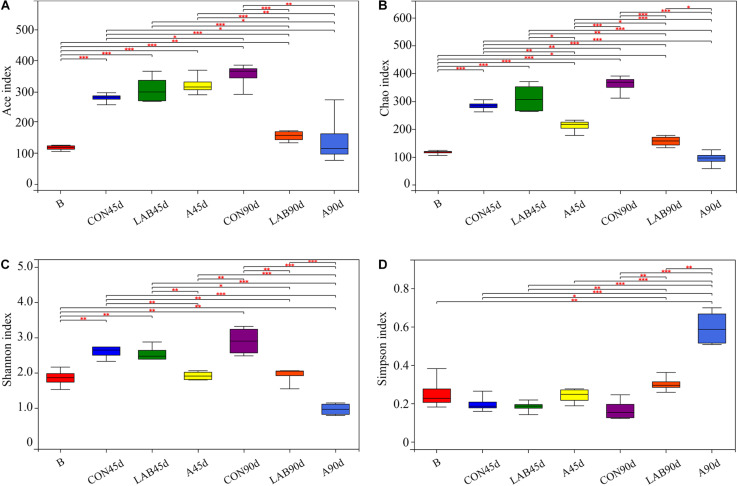
Differences in bacterial community diversity and richness between fresh corn and corn silage. **(A)** Ace index. **(B)** Chao index. **(C)** Shannon index. **(D)** Simpson index. B, fresh material; CON, control; A, organic acids; LAB, lactic acid bacteria; 45d, ensiled for 45 days; 90d, ensiled for 90 days.

In total, 1,015,896 quality-filtered sequences of 16S rRNA were acquired from 28 samples and clustered into 430 OTUs. The Good’s coverage was approximately 0.99 in all treatments (Supplementary Table S1). Ace and Chao1 increased in all treatments after ensiling except for silages treated with organic acids at day 90 (Figure 1). Prolonged ensilage enhanced Ace and Chao1 in the control groups and decreased these in the LAB and organic acid-treated groups. The Shannon index showed similar trends as Ace and Chao1. Among all silage samples, organic acid-treated silages after 90 days had the lowest Ace, Chao1, and Shannon indexes and the highest Simpson index followed by LAB-treated silages after 90 days.

**FIGURE 2:**
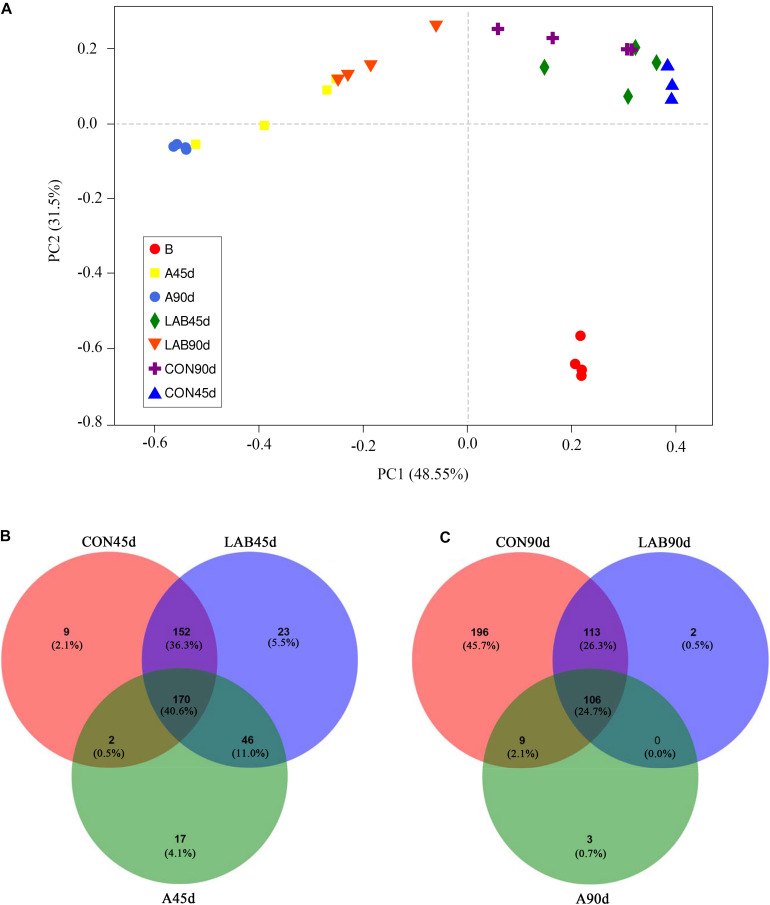
**(A)** Principle coordinate analysis (PCoA) of the bacterial community in silage samples based on Bray-Curtis distance. **(B)** Venn diagram depicting unique or common bacterial OTUs in silage samples after 45 days of ensiling. **(C)** Venn diagram depicting unique or common bacterial OTUs in silage samples after 90 days of ensiling. B, fresh material; CON, control; A, organic acids; LAB, lactic acid bacteria; 45d, ensiled for 45 days; 90d, ensiled for 90 days.

Figure 2A demonstrates the PCoA analysis of the bacterial community structure across treatments. PC1 and PC2 explained 48.55 and 31.50% of the total change, respectively. The corn silages were differed significantly from pre-ensiled corn based on ANOSIM (*R* = 0.776, *P* = 0.001). The silages of organic acid-treated corn showed a separate cluster from the control and LAB groups (*R* = 0.607, *P* = 0.001) while the microbial structure between control and LAB silages was similar (*R* = 0.145, *P* = 0.088). We also observed significant differences between ensiling for 45 and 90 days (*R* = 0.200, *P* = 0.021).

Figures 2B,C shows the number of common or unique OTUs across treatments. The organic acids groups had the lowest total number of OTUs among the three treatments after either 45 or 90 days. The unique OTUs in the additive-treated groups were reduced after 90 days compared with 45 days. In contrast, the control groups showed an increase in the number of unique OTUs from 333 to 424. Compared with 45 days of ensiling, the common OTUs were decreased in all groups from 170 to 106 after 90 days.

**FIGURE 3:**
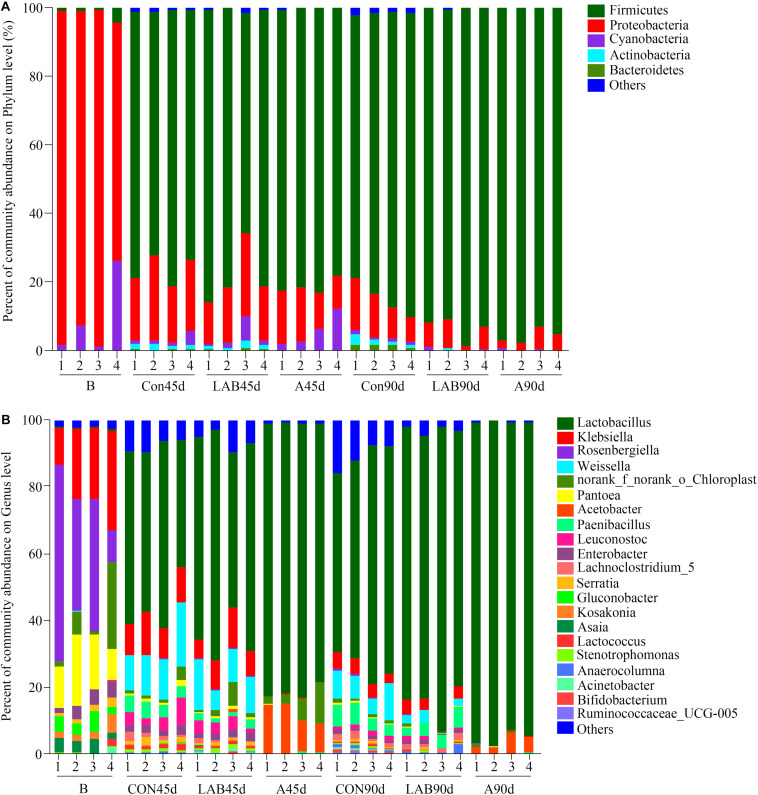
Relative abundance of bacterial composition in fresh corn and corn silage at the **(A)** phylum and **(B)** genus level. B, fresh material; CON, control; A, organic acids; LAB, lactic acid bacteria; 45d, ensiled for 45 days; 90d, ensiled for 90 days. Others represent bacteria with an abundance of <1%.

As presented in Figure 3. The dominant phyla in fresh corn were Proteobacteria (89.43%) and Cyanobacteria (8.93%) while those in silages were Firmicutes (84.48%) and Proteobacteria (11.79%) (Supplementary Table S2). The dominant genera in fresh corn were *Rosenbergiella* (35.60%) followed by *Klebsiella* (20.86%) and *Pantoea* (14.86%) (Supplementary Table S3). The most abundant genus in all silages was *Lactobacillus* and ranged from 48.43 to 95.13%. The second and third most abundant genera in control and organic acid groups after either 45 or 90 days were *Weissella* (10.60%) and *Acetobacter* (7.80%), respectively. In contrast, the second most abundant genera in LAB-treated corn silage were *Weissella* (10.45%) and *Paenibacillus* (4.15%), respectively.

**FIGURE 4:**
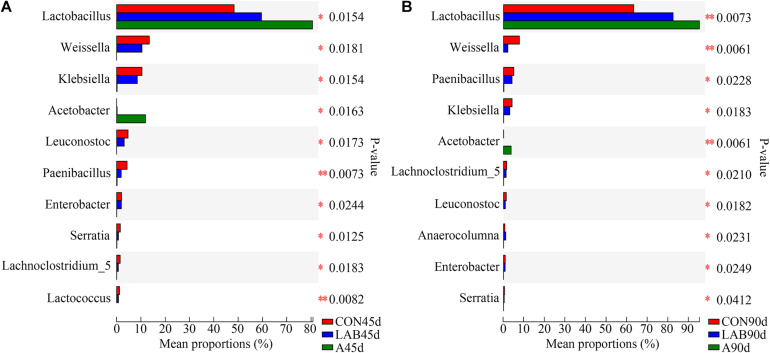
Comparison of different bacteria among the three treatments after **(A)** 45 days and **(B)** 90 days of ensiling. B, fresh material; CON, control; A, organic acids; LAB, lactic acid bacteria; 45d, ensiled for 45 days; 90d, ensiled for 90 days. ^∗^*P* < 0.05; ^∗∗^*P* < 0.01.

The proportion of *Lactobacillus* in LAB treatment was higher than in controls (59.71 vs. 48.43%), but lower than in the organic acid groups (59.71 vs. 80.74%) after 45 days (Figure 4). In contrast, the proportions of other bacteria (*Weissella*, *Klebsiella*, *Paenibacillus*, *Leuconostoc*) between LAB and control groups were similar and higher than organic acid-treated corn silage except for *Acetobacter*, which was the highest in the organic acids groups. We observed similar results after 90 days of ensilage. The proportion of *Lactobacillus* in each group was further increased with prolonged ensilage time while that of other bacteria was simultaneously reduced.

## Discussion

As expected, the DM and WSC content decreased after ensiling, which is mainly due to oxygen consumption by plant cells in silages at the early stage of ensiling followed by fermentation of WSC by microorganisms into lactic acid (Dunièrea et al., 2013). However, the dry matter concentration and recovery in organic acid-treated corn silage was lower than after LAB treatment. This result may be due to the heterofermentative fermentation process that dominates in the organic acid group, which converts lactic acid into acetic acid and CO_2_, thereby increasing dry matter loss (Wilkinson and Davies, 2013). The decreased NDF, ADF, and ADL content in silages compared with fresh material may be the result of hydrolysis of the digestible cell wall fraction by organic acids produced during ensiling (Larsen et al., 2017). The EE content of fresh material decreased after ensiling in this study and similar results have been reported in other ensiled materials such as vegetable residues (Cao et al., 2011) and mixed soybean and corn silage (Ni et al., 2018), although the precise mechanisms require further study. Compared with the CON group, the lower NDF and ADF content in LAB and organic acids groups after either 45 or 90 days of ensiling could be duo to its higher production of organic acids and thus more structural carbohydrates was hydrolyzed. In general, the acid hydrolysis of structural carbohydrates was accompanied with the release of WSC (Desta et al., 2016). However, the WSC content in LAB and organic acids groups was lower than CON group in this study, which is mainly due to the WSC content changed dynamically during the ensiling, the WSC was released by acid hydrolysis of fiber fraction, meanwhile, was used by LAB for the production of organic acids.

The pH and NH_3_-N level of silages are direct indicators of silage quality (Hashemzadeh-Cigari et al., 2014). Compared with the pH and NH_3_-N level was reported in soybean (Ni et al., 2018) and alfalfa silage (Wang et al., 2020), the low pH and NH_3_-N level in all treatment groups in this study confirmed that the corn silage was easily and successfully ensiled due to the high LAB count (5.47 log CFU/g fresh material) and WSC content (10.61% of dry matter). LAB counts of >10^5^ CFU/g fresh material (Cai et al., 1998) and a WSC content >6% dry matter (Woolford and Pahlow, 1984) have been reported to adequately ensure good fermentation of silage. In the present study, silage inoculated with LAB consisted of homofermentative lactic bacteria, which quickly ferment WSC to lactic acid in the early ensilage phase (Weinberg et al., 1993). Thus, the lactic acid content in the LAB-treated group was greater than the two other groups. The higher acetic and propionic acid concentrations in the organic acid-treated group may be caused by both the addition of organic acids and the higher level of heterofermentative fermentation. Kung and Ranjit (2001) have reported a similar increase in acetic and propionic acids for barley silage treated with propionic acid.

It is generally thought that the microbial diversity of raw materials sharply reduces after ensilage and are replaced by the LAB. However, studies so far have reported inconsistent findings. Studies in soybean and *Moringa oleifera* leaf silage indicate an increase in bacterial diversity (Ni et al., 2017; Wang et al., 2018) whereas other studies in corn silage observe a reduction (Guan et al., 2018; Xu et al., 2019). These inconsistencies may be attributed to the different ensilage time as well as different source materials. Ren et al. (2019) found that the bacterial diversity increases within 60 days and decreases after 90 days in silage of sugarcane tops. We observed a similar pattern and found an increase in bacterial diversity after 45 days paired with a decrease after 90 days in the additive-treated groups, but not in the controls. We speculate that the bacterial diversity of the control group would decrease with further prolonged ensilage time. As expected, the unique and common bacterial OTUs had the same variation trend as bacterial diversity in this study. Ren et al. (2019) suggested that not only bacterial diversity but also the unique or shared bacterial OTUs decreases with constant optimization of the silage process. Moreover, Guan et al. (2018) demonstrated that bacterial diversity sharply decreases after successful fermentation. Therefore, LAB or organic acids treatment improved the fermentation of silages evident by the reduction in microbial diversity.

The epiphytic bacterial communities of fresh materials are highly related to the source of the materials. For instance, Ni et al. (2017) reported that the dominant genera in raw soybean are *Enterobacter*, *Pantoea*, and *Serraia* while those in Moringa oleifera leaves were *Exiguobacterium*, *Acinetobacter*, and *Pseudomonas* (Wang et al., 2018). Moreover, the epiphytic bacterial communities are affected by the diverse climatic conditions of different areas. Guan et al. (2018) found that rainfall and humidity had a significant impact on these bacteria in corn. Guan et al. (2020) observed that *Leuconostoc*, *Klebsiella*, and *Lactococcus* are the main bacteria on corn while *Rosenbergiella*, *Klebsiella*, and *Pantoea* were the dominant genera in our current study. Interestingly, previous studies consistently report that the relative abundance of *Lactobacillus* is low in fresh material and expands after ensilage (Keshri et al., 2018; Guan et al., 2020).

It has been proven that the additives affect the quality of silage by changing the bacterial profile (Ni et al., 2017; Liu et al., 2019). In this study, LAB treatment resulted in higher lactic acid content, lower pH, and similar acetic acid concentration compared to controls, implying that the fermentation pattern in LAB-treated corn silage was homofermentative and could be explained by the homofermentative nature of the LAB used in our experiments. Moreover, the higher level of *Lactobacillus* in the LAB group after 45 and 90 days confirmed the successful colonization and propagation of the added LAB in corn silages. On the contrary, the addition of organic acids typically leads to heterofermentative fermentation evident by the high abundance of *Acetobacter* and acetic acid concentration. *Acetobacter* dominance has also been reported in both small-scale laboratory (Guan et al., 2020) and farm bunker-silo corn silage (Guan et al., 2018), although its presence is somewhat controversial. On one hand, it metabolizes sugars and alcohol to acetic acid that potentially inhibit the growth of fungi and thus improve the aerobic stability of silage (Nanda et al., 2001; Kristensen et al., 2010). On the other hand, high acetic acid content is related to large dry matter losses and could lead to a reduction in dry matter intake (Guan et al., 2018), although other studies did not observe such an effect (Kristensen et al., 2010; Muck et al., 2018). Thus, more research is required to determine the impact of high *Acetobacter* and acetic acid content in silage on the intake of animals. Unexpectively, silage treated with LAB did not exhibit high abundance of *Lactobacillus* compared with organic acid group, which was likely because the organic acids inhibited undesired bacteria and thus provided optimal conditions for rapid LAB growth and domination in the silage (Kung et al., 2003). Interestingly, the addition of organic acids in our study decreased undesirable microorganisms such as *Klebsiella*, *Paenibacillus*, and *Enterobacter*. *Klebsiella* are gram-negative facultative anaerobes that cause multiple diseases (Podschun and Ullmann, 1998). *Paenibacillus* in silages is one of the primary sources of spore-forming contamination in farms (Coorevits et al., 2008). Spore formers may subsequently contaminate the milk via the cow’s diet and produce toxins and spoilage enzymes, thus exerting harmful effects on food safety and product quality (Borreani et al., 2013). *Enterobacteria* is undesirable due to its ability to ferment acetic acid, resulting in a loss of nutrition (Ni et al., 2017). Therefore, compared with LAB, organic acids have a greater advantage in accelerating the domination of *Lactobacillus* in corn silage and reducing undesirable microorganisms.

## Conclusion

We demonstrated significant changes in bacterial composition and diversity of corn silage treated with different additives. LAB improved the fermentation of corn silage by increasing the lactic acid concentration and decreasing pH in the early phase of fermentation. In contrast, organic acids tended to promote heterofermentative fermentation by increasing acetic acid concentration and the relative abundance of *Acetobacter*. In addition, compared with LAB, organic acids were more advantageous for accelerating *Lactobacillus* expansion and reducing undesirable microorganisms in silages.

## Data Availability Statement

The raw sequence reads were deposited into the NCBI Sequence Read Archive (SRA; http://www.ncbi.nlm.nih.gov/Traces/sra/) database under the accession numbers SRR12972050 to SRR12972077.

## Author Contributions

FJ, HC, and ES designed the study and wrote the manuscript. FJ, DL, CW, YW, HS, and WA performed the experiments. FJ and ES analyzed the data. All authors reviewed the manuscript.

## Conflict of Interest

The authors declare that the research was conducted in the absence of any commercial or financial relationships that could be construed as a potential conflict of interest.
